# Implementation of less invasive surfactant administration in clinical practice—Experience of a mid-sized country

**DOI:** 10.1371/journal.pone.0235363

**Published:** 2020-07-06

**Authors:** Tomasz Szczapa, Roman Hożejowski, Paweł Krajewski, on behalf of the Study Group

**Affiliations:** 1 Department of Neonatology, Poznan University of Medical Sciences, Poznan, Poland; 2 Medical Department, Chiesi Poland, Warsaw, Poland; 3 Department of Neonatology, University Center for Mother and Newborn’s Health, Warsaw, Poland; Centre Hospitalier Universitaire Vaudois, FRANCE

## Abstract

**Objective:**

There are differences in the adoption rates of less invasive surfactant administration (LISA) worldwide. We aimed to describe and analyze the process of LISA introduction at the country level.

**Methods:**

A standardized training program (33 courses covering >500 neonatologists) was followed by a cohort study. Data regarding consecutive LISA procedures were acquired over 12 months in 31 tertiary neonatal centers, using a dedicated on-line platform.

**Results:**

Of 500 LISA procedures, 75% were performed by specialists and 25% by residents. The mean percentage share of LISA in all surfactant therapies was 24%, which represents a 6-fold increase compared to previous years. After 12 months, 76% of the procedures were rated “easy/very easy” vs 59% at baseline (*p*<0.05). Surfactant re-treatment rate was 15%. Twenty-three percent of infants required mechanical ventilation within 72 hours of life. Oxygen desaturation and surfactant reflux were the most frequent complications. Unlike previous reports describing exclusive use of nasal continuous positive airway pressure (nCPAP) during LISA, majority of procedures (63%) were carried out using nasal intermittent positive pressure ventilation (NIPPV) or Bilevel Positive Airway Pressure (BiPAP). Efficacy of LISA with NIPPV or BiPAP was not significantly different from that with nCPAP (22.4% vs 24.5% of cases requiring intubation). Ventilation was provided with nasal cannulas or nasal masks (90%) and rarely with “RAM” cannulas or nasopharyngeal tubes. Rigid catheters were preferred (88.4%); tracheal insertion was successful at first attempt in 87% of cases. Majority of infants (79%) received no premedication prior to the procedure and almost all were given caffeine citrate. Median time of instillation was 1.5 minutes.

**Conclusions:**

The LISA procedure does not appear to be technically difficult to master. Training combining theory with practical exercises is an efficient implementation strategy. Variations in adoption rates indicate the need for additional, more personalized teachings in some centers.

## Introduction

The approach to ventilatory support in preterm infants with respiratory distress syndrome (RDS) has evolved into a more frequent use of non-invasive modes, resulting in improved clinical outcomes and a potential reduction of hospitalization costs [1–3]. Following this trend, techniques of less invasive techniques surfactant administration (LISA) have become increasingly popular. At present, LISA techniques are an integral part of the RDS treatment bundle in patients who are at risk of failure of non-invasive ventilation [4–7]. Since the latest update of the European Consensus Guidelines on the Management of RDS in 2019, LISA is considered the preferred method of surfactant delivery, provided that clinicians have adequate experience [7]. With increasing evidence of clinical benefits, LISA is becoming more widespread; however, its adoption is quite different in various regions of the world. Based on recent reports, routine use of this method is declared by only 8% of neonatologists in the USA [8] and 11% of neonatal units in England [9]. In other countries, such as Germany or Spain, LISA already seems to be the dominant method of surfactant administration [6, 10]. Data regarding Eastern Europe are lacking, but studies conducted in Poland in 2014 and 2015 have shown that LISA is even less frequently used–in approximately 4% of neonates ≤32 weeks’ gestation [11, 12].

Despite being perceived as a relatively simple procedure, LISA requires certain skills. To promote this less invasive approach to surfactant delivery in Poland–a country with initial low adoption rates–a series of standardized LISA trainings were carried out across tertiary neonatal centers. These were followed by a dedicated study to monitor the implementation of LISA.

The aim of this multicenter prospective cohort study was to assess the process of LISA adoption and to describe its use in everyday practice.

## Methods

A run-in phase preceding initiation of the study included a nationwide series of standardized LISA workshops in level-3 NICUs. The trainings comprised unified theoretical lectures and hands-on exercises of LISA. During the sessions, realistically proportioned 25-week preterm manikins (Premature Anne, Laerdal) were used along with dedicated catheters (LISAcath, Chiesi Farmaceutici, Italy), standard laryngoscopes and various nCPAP interfaces. Over 33 courses, more than 500 neonatologists and neonatal residents were trained by six neonatal experts who had previous experience with LISA. Starting in February 2018, data on LISA applications were prospectively collected in 31 of 64 (48%) tertiary referral NICUs during a 12-month period. The study addressed four aspects: (1) technical practicalities of the procedure, (2) ease of learning, (3) LISA uptake in the study centers and (4) possible complications during LISA (oxygen desaturation, surfactant reflux, bradycardia, apnea, rescue intubation or unilateral surfactant deposition). Data was collected using a dedicated on-line reporting platform. The process of data acquisition and analysis was carried out by a third-party, independent contract research organization.

Ease of learning was analyzed based on self-assessments of LISA difficulty by the proceduralists. Each procedure was rated on a six-grade scale, encompassing the following categories: “very easy”, “easy”, “not too difficult”, “difficult”, “very difficult”, and “impossible to perform”. Combined “easy” and “very easy” rates were assessed on a quarterly basis over the period of the study.

LISA uptake was defined as the percentage share of LISA in all surfactant therapies in the study centers.

The study protocol did not interfere with the routine therapeutic process for neonatal RDS, which reflected the standard care in the study centers, e.g. the choice of surfactant preparation (poractant alfa and beractant were available in Poland during the study period). This also applied to all medicinal products, which were prescribed in the usual manner. The Bioethical Committee of Warsaw Medical University approved the study protocol (ref. no. AKBE/213/2017), and all parents provided written consent to all therapeutic and diagnostic procedures, according to local law and practices.

### Statistics

A mixed-effect logistic regression model was used to compare the ease of the LISA procedure across consecutive quarters of the study. The fixed-effect factors were the calendar time of the procedure (corresponding quarter) and the qualifications of the performer (specialist vs resident), while the study center was set as a random effect. Quarterly odds ratios with corresponding 95% confidence intervals were calculated versus Q1 2018 (baseline). Descriptive statistics were provided for all study parameters. A *p*-value less than 0.05 was considered significant.

## Results

From February 2018 to February 2019, data on 500 LISA procedures were collected. The clinical characteristics of the study cohort are presented in Table 1.

**Table 1:** Patients’ demographic data and interventions in the delivery room.

Variable	
Sex (male)	274 (55%)
Antenatal steroids	392 (78%)
Gestational age	
≤28 weeks	169 (34%)
29–32 weeks	214 (43%)
>32 weeks	116 (23%)
Cesarean delivery	453 (90.6%)
Apgar 5 min. (median, IQR)	9 (7–8)
Birth weight (g)	
Median (IQR)	1330 (990–1721)
Range	450–3630
Supplemental oxygen in the DR	420 (84%)
Maximum FiO_2_ in the DR (median, IQR)	0.35 (0.30–0.50

DR, delivery room.

### Performance of the LISA procedure

Most procedures (98.8%) were performed in the NICUs. LISA was mainly the domain of specialists, but neonatal trainees had a considerable 25% share. Intubation proficiency (score 9 or 10 on a 0–10 scale) was declared by only 53% of LISA performers.

In the majority of infants (392/500; 79%), no premedication was administered. Atropine was used in only 12 infants (2.4%). The surfactant (SF) of choice was poractant alfa, used in 98% of infants, at a median (IQR) dose of 192 (158–200) mg/kg BW. Beractant was given to the remaining 2% of infants. Seventy-six infants (15.4%) required SF retreatment. Decisions regarding retreatment were made at physician’s discretion, according to local protocols. Of those infants who required two doses of SF, 26 (41%) received the second dose using LISA again. Of those babies who required three doses of SF (n = 12), LISA was used in 50% of babies during the first retreatment and in 1 infant during the second retreatment.

All but 4 babies in the studied cohort received caffeine citrate, either prior to surfactant treatment (63%) or concomitantly with surfactant. Further details on LISA are summarized in Table 2.

**Table 2:** Performance of LISA procedure. Data are n (%) or median (IQR).

Variable		
Age at LISA (h)		2.1 (0.8–6.7)
FiO_2_ prior to LISA		0.40 (0.35–0.50)
SpO_2_ prior to LISA		90 (87–93)
Performer’s experience in intubation (0 = none, 10 = expert)		
	Consultant	9 (8–10)
	Resident	6 (4–7)
Type of catheter		
	LISAcath	430 (86%)
	Nasogastric tube	35 (7%)
	Vascular catheter	12 (2.4%)
	Suction catheter	6 (1.2%)
	Other	17 (3.4%)
Non-invasive respiratory support*		
	nCPAP	201 (40%)
	BiPAP	193 (39%)
	NIPPV	119 (24%)
	HFNC	1 (0.2%)
	NHFOV	1 (0.2%)
Type of interface		
	Nasal cannula	277 (55%)
	Nasal mask	177 (35%)
	RAM cannula	38 (7.6%)
	Nasopharyngeal tube	8 (1.6%)
Duration of surfactant instillation (min:s)		
	Median (IQR)	1:30 (1:00–2:00)
	Range	0:10–10:00
No. of attempts to insert the catheter		
	1 attempt	437 (87%)
	2 attempts	56 (11%)
	3 attempts	6 (1.2%)
	4 attempts	1 (0.2%)

* Percentages do not add up to 100 because in some patients the mode of ventilatory support was changed during the procedure.

BiPAP, Bilevel Positive Airway Pressure; HFNC, high flow nasal cannula; NHFOV, non-invasive high frequency oscillatory ventilation.

### Difficulty of LISA and the learning process

**Fig 1:**
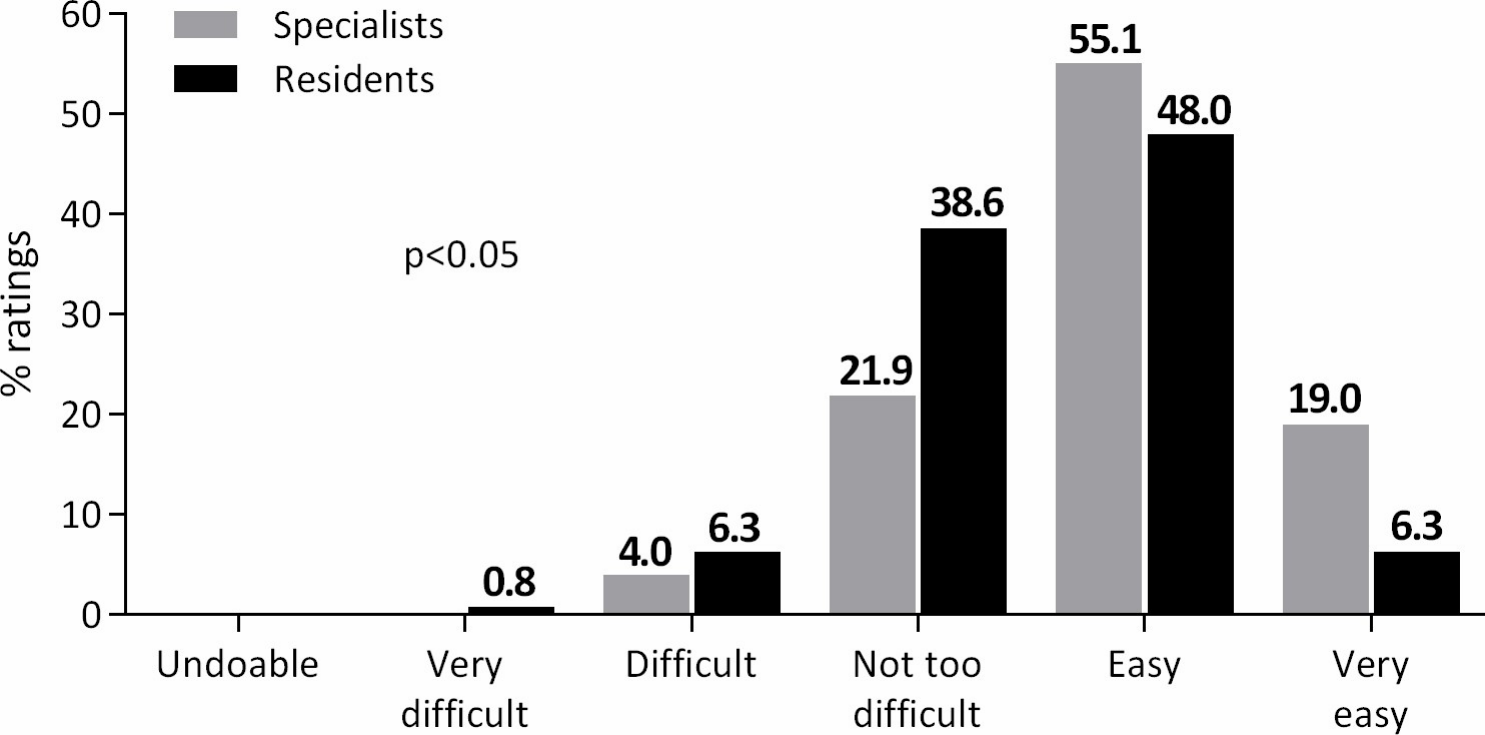
Ratings of the difficulty of the LISA procedure according to the qualification of the proceduralist (specialists vs residents).

LISA was rated as “very difficult” in only one infant by a resident. Overall, in 345 cases (69%), the procedure was rated “easy” or “very easy”. There was a slight yet statistically significant (*p*<0.05) difference in ratings between residents and specialists. As shown in Fig 1, residents rated LISA as difficult more often. In the mixed-effects model, when performed by a specialist, the procedure was associated with a significantly higher chance of being rated “easy” or “very easy” (OR 2.67, 95% CI 1.59–4.54).

**Fig 2:**
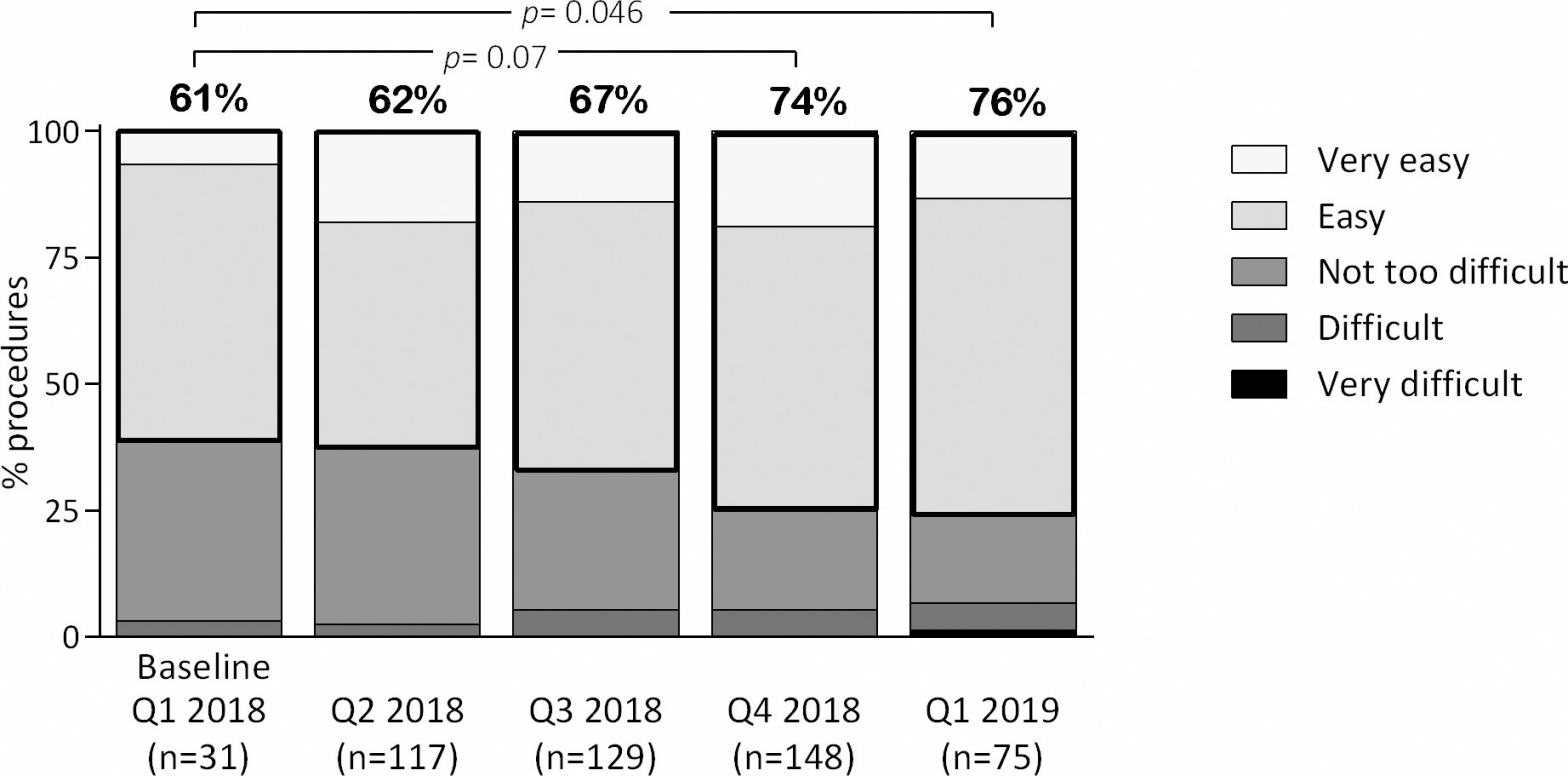
Assessment of difficulty of the LISA procedure over consecutive quarters of the study period. The figures above each column indicate the proportion of combined “easy” and “very easy” grades.

During the study, the percentage of combined “easy” or “very easy” ratings increased from the baseline value of 59% to a maximum of 76% after 12 months (Fig 2). The last quarter of the study (Q1 2019) was associated with significantly higher odds for combined “easy” or “very easy” ratings compared to the baseline quarter (OR 2.87, *p* = 0.046).

### Complications during LISA

There were 255 adverse events (AEs) reported, affecting 184 patients in total. The most common AE was oxygen desaturation (22%), which involved a SpO_2_ decrease from a median (IQR) of 90% (87–93%) before LISA to 85% (77–90%) during LISA (*p*<0.0001, Wilcoxon test). Reported complications were summarized in Table 3.

**Table 3:** Adverse events during LISA.

Adverse event	n (%)
Oxygen desaturation	110 (22%)
Surfactant reflux	94 (18.8%)
Bradycardia	21 (4.2%)
Apnea	19 (3.8%)
Need for rescue intubation	6 (1.2%)
Unilateral surfactant deposition	2 (0.4%)

### LISA uptake in the study centers

The percentage share of LISA in all surfactant therapies was on average 24%, but with considerable differences in adoption rate between the study sites (range 3.5–61%). A LISA share of <10% was seen in 4 centers, between 10 and 30% in 16 centers, and >30% in 11 centers.

### LISA efficacy

Out of 500 infants, 114 (22.8%) required intubation and invasive ventilation within 72 hours from birth, which was considered treatment failure. The failure rates depended on gestational age and varied from a maximum of 69% at 24 weeks to a minimum of 4% at 34 weeks. No significant differences in LISA efficacy were found depending on whether nCPAP vs NIPPV or BiPAP were used (24.5% vs 22.4% of cases requiring intubation within 72 hours; p = 0.66).

## Discussion

To our knowledge, this is the first study exploring the process of a practically *de novo* implementation of LISA in everyday practice at a country level. LISA is an interesting example of a medical technology that is adequately backed by medical evidence showing its superiority over SF administration via endotracheal tube [13, 14], yet its implementation remains problematic at bedside, and adoption rates are disappointing in many countries [8, 9]. This study included an assessment of the learning process and the rate of LISA adoption—aspects that have not been investigated before.

LISA is usually perceived as a highly specialized procedure that should be performed by experienced specialists. It was therefore unexpected that as many as 1/4 of the procedures had been performed by neonatal trainees and that only half of the performers declared intubation proficiency. Nevertheless, the high success rate of the first attempt of catheter placement shows that technically, LISA was relatively uncomplicated to perform. This was confirmed by the difficulty assessments, where the percentage of combined “easy” and “very easy” scores was high. In contemporary neonatology, intubation opportunities for residents are relatively rare [15]. As intubation experience plays a vital role, unsurprisingly, the proportion of neonatal trainees perceiving LISA as easy or very easy was almost 20% lower compared to neonatal consultants. However, confidence regarding LISA grew with time. The proportion of “easy” and “very easy” scores increased by a noticeable 17 percentage points within a year.

Perceptions of the LISA difficulty might also be influenced by the equipment used. While feeding tubes have been most commonly chosen for LISA in some European countries, as shown in the 2017 survey [5] and in the report from Nordic countries [16], the use of soft nasogastric and suction catheters was reported in only 8% of patients in our cohort. Most procedures were performed using more rigid catheters, which are easier to handle and do not require Magill forceps. The options included LISAcath or vascular catheters, which had been used in almost 90% of infants. LISAcath was previously shown to be the most commonly used catheter in a recent survey from the UK [17, 18]. Semirigid vascular catheters are also preferred by Spanish doctors [10]. Based on a manikin study, semirigid or stylet-guided vs flexible catheters allow faster tracheal insertion and are perceived as easier to use, although they have not been compared in a randomized study [19].

In the studied cohort, the median dose of poractant alfa was 192 mg/kg, which is very close to the recommended 200 mg/kg. This might have an impact on the low rate of surfactant retreatment (15%), compared to an approximately twice-higher rate of retreatment (28%) in the meta-analysis by Aldana-Aguirre et al. [20].

LISA was used in 41% of infants requiring SF retreatment. This rate is similar to the 39% rate reported in a recent study by Janssen et al. [21]. Nonetheless, we find it to be quite high for the implementation period. As the method was new and still being learned, a lower confidence in LISA could be expected.

Unlike other reports where standard nasal CPAP was used with LISA [21–24], 63% of the procedures were carried out using BiPAP or NIPPV. While the potential advantages of BiPAP over CPAP remain uncertain, NIPPV proved to be more effective than nCPAP in reducing respiratory failure and the need for mechanical ventilation among preterm infants with RDS [7, 25, 26]. In our cohort the effectiveness of LISA with NIPPV or BiPAP did not differ significantly from that of LISA with nCPAP, however this issue could be investigated more thoroughly in the future.

Nasal cannulas or nasal masks were the interfaces used in 90% of our patients. Since nasal masks have a lower intrinsic resistance than short binasal prongs, their use during LISA might potentially influence their clinical effectiveness [27]. The type and size of the interface is essential for the ease of performing the procedure, as it may affect the laryngoscopic view. Therefore, smaller, low-profile interfaces are preferred by some practitioners. Less than 2% of the procedures in this study were carried out with respiratory support via a nasopharyngeal tube and only 8% via a RAM cannula. The application of a RAM cannula might facilitate laryngoscopy, but its high resistance leads to a greater drop in delivered airway pressure compared to the circuit pressure set. Hence, it is uncertain whether this interface is the optimal choice for LISA [27].

The median duration of surfactant instillation was 1.5 minutes, which is within the range recommended by experts [28]. However, in the European survey, as much as 46% of respondents declared surfactant administration within less than 1 minute [5], which contrasts with only 25% of infants in the studied cohort who were instilled surfactant that fast. This might be explained by greater caution in performing the procedure that was perceived as new and still in the process of learning.

Approximately 80% of the patients received no premedication, and other studies confirmed large variability in approaches to LISA premedication [5, 6, 28]. Despite potential usefulness of atropine [28], only 2.4% of infants in the analyzed group received this drug.

LISA was an effective procedure for most newborns in the study. The proportion of treatment failures in relation to the mean gestational age (GA) was similar to data from the available literature and showed a dependence on GA [6, 20].

The overall rate of complications was relatively low, considering findings of the European survey by Klotz et al. [5], where no adverse effects during the procedure were reported by only 22% of the respondents. Also, majority of symptoms had mild to moderate severity. The incidence of oxygen desaturation (22%) was somewhat higher compared to 17% in the randomized controlled study of Kanmaz et al. [22], but this might be attributable to the multicentric design, where not all NICUs and performers had equal experience. On the other hand, the reflux rate (19%) was almost identical to that of the meta-analysis of six LISA studies, where this complication was observed in 20% of infants [20].

The LISA implementation process in Poland seems successful, as the application rate has increased six-fold in relation to available baseline data from previous Polish studies [11, 12]. However, these results must be taken with caution due to study limitations. Despite the significant number of neonatal units involved, data obtained may not be representative for all Polish centers and LISA uptake varied largely between them. Nonetheless, the mean use of LISA (24%) was higher than in the USA [8] or England [9], although lower than in Nordic countries [16], Germany [6] or Spain [10]. Comparison of the available literature data may be ambiguous since it is not obvious how the percentage of “units which use LISA” translates into the actual percentage of infants treated with LISA. In the reported study, patients’ data were prospectively collected from nearly half of all tertiary NICUs in the country, which gives a reliable picture.

The observed rise in LISA uptake in Polish NICUs was likely associated with the educational program and high number of physicians trained. Experiences from this process indicate that for the best effects, such training must combine theoretical and practical instruction, using realistic manikins, video material recorded at bedside and videolaryngoscopy if available [28]. Observed variations in LISA adoption rates show that changes in well-established treatment patterns may be challenging in some centers. In such cases, repeated courses might be considered, and the training program can be further optimized.

In summary, the results of our study show that the LISA procedure is technically relatively easy to learn. Extensive training that integrates theory with practical exercises has proven to be an efficient way to facilitate its introduction.

## Supporting information

S1 File (PDF)
