# Supplementary material for: Implementation of less invasive surfactant administration in clinical practice—Experience of a mid-sized country
Source: PLoS One. 2020 Jul 6;15(7):e0235363. doi: 10.1371/journal.pone.0235363 (PMC7337349; doi:10.1371/journal.pone.0235363)
Supplement: S1 File — (PDF) [file pone.0235363.s001.pdf]

Electronic CRF items with descriptions in English

Date
CRM ID
Investigator
Patient ID
INC_01. Premature infant with established Respiratory Distress Syndrome (RDS) or at risk for RDS
INC_02. Presence of spontaneous breathing
INC_03. Decision of the attending physician to administer surfactant using LISA/MIST method
EXC_01. Newborn with clinically significant maxillo-facial, tracheal or known pulmonary malformations
EXC_02. The need for intubation and mechanical ventilation in the Delivery Room
IEYN. Patient can participate in the study
DN.NOTES.txt. Notes
DN.DU. Birth date
DN.DU. Time of birth
DN.PL. Sex
DN.WC.TYG. Gestational age (weeks)
DN.WC.DNI. Gestational age (days)
DN.STEROID. Antenatal corticosteroids
DN.SPOSURO. Mode of delivery
DN.CN. Multiparity
DN.UMC.int. Birth weight
APGAR.1MIN.99. APGAR not done
APGAR.1MIN.01. Skin
APGAR.1MIN.02. Pulse rate
APGAR.1MIN.03. Reflex
APGAR.1MIN.04. Muscle Tone
APGAR.1MIN.05. Respiratory effort
APGAR.1MIN.06. APGAR result 1 min
APGAR.5MIN.01. Skin
APGAR.5MIN.02. Pulse rate
APGAR.5MIN.03. Reflex
APGAR.5MIN.04. Muscle Tone
APGAR.5MIN.05. Respiratory effort
APGAR.5MIN.06. APGAR result 5 min
SNSP.OT. Thermal protection (polyethylene bag)
SNSP.ORDC. Positive pressure lung inflation
SNSP.ORDC.01.pw. Self-inflating bag
SNSP.ORDC.02.pw. Neopuff
SNSP.ORDC.03.pw. Other
SNSP.ORDC.03.txt. Other
SNSP.TLTRP. Oxygen therapy in the Delivery Room
SNSP.TLTRP.POCZ.float. Initial FiO2
SNSP.TLTRP.MAKS.float. Maximum FiO2
WYKLISA.MSCWYK. LISA procedure localization
WYKLISA.LEKARZ. LISA proceduralist
WYKLISA.DOSW. Experience in intubation [0-10, where 0=none, 10=expert]
WYKLISA.RODZCEW. Type of catheter
WYKLISA.RODZCEW.txt. Type of catheter - other
WYKLISA.KLMAG. Use of Magill forceps
WYKLISA.PREMED. Premedication
WYKLISA.PREMED.01.pw. Sublingual glucose
WYKLISA.PREMED.02.pw. Atropine

WYKLISA.PREMED.03.pw. Fentanyl
WYKLISA.PREMED.04.pw. Sufentanil
WYKLISA.PREMED.05.pw. Morphine
WYKLISA.PREMED.06.pw. Ketamine
WYKLISA.PREMED.07.pw. Propofol
WYKLISA.PREMED.08.pw. Midazolam
WYKLISA.PREMED.09.pw. Other
WYKLISA.PREMED.09.txt. Other
WYKLISA.NIEINWODD.TRYB.01.pw. nCPAP
WYKLISA.NIEINWODD.TRYB.02.pw. BiPAP
WYKLISA.NIEINWODD.TRYB.03.pw. NIPPV (Nasal intermittent positive pressure ventilation)
WYKLISA.NIEINWODD.TRYB.04.pw. HFNC (High Flow Nasal Cannula)
WYKLISA.NIEINWODD.TRYB.05.pw. Other
WYKLISA.NIEINWODD.TRYB.05.txt. Other
WYKLISA.NIEINWODD.RODZAJ. Type of interface
WYKLISA.NIEINWODD.RODZAJ.KANIULE. Type of interface - nasal
WYKLISA.SZYBK. Duration of surfactant instillation (min:s)
WYKLISA.OCTRUD. Difficulty of LISA procedure: self-assessment (ordinal scale)
WYKLISA.LICZBAPROB.int. Number of attempts to insert a catheter into the trachea
SIK.NAZSURF. Surfactant generic name
SIK.OBJDWKPOCZ. Initial surfactant dose volume [ml]
SIK.DATAPOD. Date of surfactant instillation
SIK.CZASPOD. Time from birth to surfactant [hours]
SIK.POW24H. Time from birth to surfactant >24h
SIK.CZASPOD.WSK.txt. Reason for surfactant instillation >24h
SIK.PRZED.FiO2. FiO2 prior to surfactant
SIK.PRZED.SpO2. SpO2 prior to surfactant
SIK.DTKOFNA. Data of caffeine citrate administration
SIK.TMKOFNA. Time of surfactant instillation
BIT.REFLUKS. Surfactant reflux
BIT.SURFPŁC. Unilateral surfactant deposition
BIT.BRADKRD. Clinically significant bradycardia
BIT.BRADKRD.ZWZ. Clinically significant bradycardia - relationship to drug
BIT.BRADKRD.NOBJ. Clinically significant bradycardia - symptom severity
BIT.BEZDECH. Clinically significant apnea
BIT.BEZDECH.ZWZ. Clinically significant apnea - relationship to drug
BIT.BEZDECH.NOBJ. Clinically significant apnea - symptom severity
BIT.DESTRC. Clinically significant oxygen desaturation
BIT.DESTRC.ZWZ. Clinically significant oxygen desaturation - relationship to drug
BIT.DESTRC.NOBJ. Clinically significant oxygen desaturation - symptom severity
BIT.DESTRC. Clinically significant need for rescue intubation
BIT.DESTRC.ZWZ. Clinically significant need for rescue intubation - relationship to drug
BIT.DESTRC.NOBJ. Clinically significant need for rescue intubation - symptom severity
BIT.IZN. Other adverse events
BIT.IZN.txt. Other adverse events
BIT.IZN.ZWZ. Other adverse events - relationship to drug
BIT.IZN.NOBJ. Other adverse events - symptom severity
BIT.SATUR.float. Lowest saturation (SpO2) during LISA/MIST
BIT.STEZENIE. Highest oxygen concentration (FiO2) during LISA/MIST
FLT. At least one AE reported
ZN.PAC.PLEC. Sex
ZN.PAC.MASA.int. Body weight

ZN.DATA.02.DATE. Date of event - Bradycardia
ZN.CIĘZKIEZN.02. Serious adverse event - Bradycardia
ZN.DATA.03.DATE. Date of event - Apnea
ZN.CIĘZKIEZN.03. Serious adverse event - Apnea
ZN.DATA.04.DATE. Date of event - Desaturation
ZN.CIĘZKIEZN.04. Serious adverse event - Desaturation
ZN.DATA.06.DATE. Date of event - Clinically significant need for rescue intubation
ZN.CIĘZKIEZN.06. Serious adverse event - Clinically significant need for rescue intubation
ZN.OPIS.01.txt. Description of other adverse events
ZN.DATA.07.DATE. Date of event - Other
ZN.CIĘZKIEZN.07. Serious adverse event - Other
ZN.KLAS.01.pw. Death
ZN.KLAS.02.pw. Life-Threatening
ZN.KLAS.03.pw. Permanent or significant disability or impairment
ZN.KLAS.04.pw. Hospitalization or its extension
ZN.KLAS.05.pw. Others, which the physician considers to be severe
ZN.KLAS.06.pw. Not applicable
ZN.STATPRZG.txt. Statistical number of the cause of death
ZN.LECZSZP. Hospital treatment
ZN.WYNZN. Result of adverse event
ZN.STLEKI.NLEKU.02. Drug name
ZN.STLEKI.PODEJ.02. Drug suspected of causing symptoms
ZN.STLEKI.DDOB.02. Daily dose
ZN.STLEKI.DRPOD.02. Route of administration
ZN.STLEKI.DATAR.02. Start date of administration
ZN.STLEKI.DATAZ.02. End date of administration
ZN.STLEKI.PUNSC.02. Cause of use or statistical number of the disease
ZN.STLEKI.NLEKU.03. Drug name
ZN.STLEKI.PODEJ.03. Drug suspected of causing symptoms
ZN.STLEKI.DDOB.03. Daily dose
ZN.STLEKI.DRPOD.03. Route of administration
ZN.STLEKI.DATAR.03. Start date of administration
ZN.STLEKI.DATAZ.03. End date of administration
ZN.STLEKI.PUNSC.03. Cause of use or statistical number of the disease
ZN.STLEKI.NLEKU.04. Drug name
ZN.STLEKI.PODEJ.04. Drug suspected of causing symptoms
ZN.STLEKI.DDOB.04. Daily dose
ZN.STLEKI.DRPOD.04. Route of administration
ZN.STLEKI.DATAR.04. Start date of administration
ZN.STLEKI.DATAZ.04. End date of administration
ZN.STLEKI.PUNSC.04. Cause of use or statistical number of the disease
ZN.STLEKI.NLEKU.05. Drug name
ZN.STLEKI.PODEJ.05. Drug suspected of causing symptoms
ZN.STLEKI.DDOB.05. Daily dose
ZN.STLEKI.DRPOD.05. Route of administration
ZN.STLEKI.DATAR.05. Start date of administration
ZN.STLEKI.DATAZ.05. End date of administration
ZN.STLEKI.PUNSC.05. Cause of use or statistical number of the disease
ZN.STLEKI.NLEKU.06. Drug name
ZN.STLEKI.PODEJ.06. Drug suspected of causing symptoms
ZN.STLEKI.DDOB.06. Daily dose
ZN.STLEKI.DRPOD.06. Route of administration

ZN.STLEKI.DATAR.06. Start date of administration
ZN.STLEKI.DATAZ.06. End date of administration
ZN.STLEKI.PUNSC.06. Cause of use or statistical number of the disease
ZN.DODINF.txt. Additional information
ZN.DOS.NAZW.txt. First and last name of the person reporting
ZN.DOS.SPEC.txt. Medical specialization
ZN.DOS.TEL.int. Phone number
ZN.DOS.FAX.int. Fax
WL.SURFIKOF.SURF. Total number of surfactant doses
WL.SURFIKOF.KOFN.float. The total duration of treatment with caffeine citrate
WL.SURFIKOF.DAWSURF.01.01. Method of surfactant administration
WL.SURFIKOF.DAWSURF.01.02. Time of extubation
WL.SURFIKOF.DAWSURF.02.01. Method of surfactant administration
WL.SURFIKOF.DAWSURF.02.02. Time of extubation
WL.SURFIKOF.DAWSURF.03.01. Method of surfactant administration
WL.SURFIKOF.DAWSURF.03.02. Time of extubation
WL.SURFIKOF.DAWSURF.04.01. Method of surfactant administration
WL.SURFIKOF.DAWSURF.04.02. Time of extubation
WL.SURFIKOF.DAWSURF.05.01. Method of surfactant administration
WL.SURFIKOF.DAWSURF.05.02. Time of extubation
WL.WENTMECH. Need for intubation and mechanical ventilation during hospitalization
WL.WENTMECH.PON72H. Within 72h since birth
WL.WENTMECH.DTROZP. Start date of mechanical ventilation
WL.WENTMECH.TMROZP. Start time of mechanical ventilation
WL.WENTMECH.LCZAS. Duration of mechanical ventilation - days
WL.WENTMECH.LCZAS. Duration of mechanical ventilation - hours
WL.WENTMECH.LCZAS. Duration of mechanical ventilation - minutes
WL.WENTMECH.HFOV. Use of High Frequency Oscillatory Ventilation (HFOV)
WL.WENTNINW.TRYB.01.pw. nCPAP
WL.WENTNINW.TRYB.02.pw. Biphasic positive airway pressure (BiPAP)
WL.WENTNINW.TRYB.03.pw. NIPPV/synchronized NIPPV
WL.WENTNINW.TRYB.04.pw. High-flow nasal cannulas (HFNC)
WL.WENTNINW.LCZAS. Duration of non-invasive ventilation - days
WL.WENTNINW.LCZAS. Duration of non-invasive ventilation - hours
WL.WENTNINW.LCZAS. Duration of non-invasive ventilation - minutes
WL.TLENO.SUPL. Oxygen supplementation (> 21%) for at least 28 days
WL.TLENO.ZAPOTRZ. Oxygen demand at 36 weeks corrected age (or at the time of discharge):
WL.POWWCZ.RETIN. Retinopathy of prematurity
WL.POWWCZ.KOD. Intraventricular hemorrhage
WL.POWWCZ.KOD.CK. Severe intraventricular hemorrhage
WL.POWWCZ.LOK. Periventricular leukomalacia
WL.POWWCZ.PPT. Persistent ductus arteriosus requiring treatment
WL.POWWCZ.PPT.01.pw. Pharmacological treatment
WL.POWWCZ.PPT.02.pw. Ligation
ZO.OODZ.POW. Reason for hospital discharge
ZO.OODZ.DATA. Date of leaving the hospital ward
ZO.OODZ.GODZ. Time of leaving the hospital ward
ZO.UWAGI.txt. Comments
